# Poly (Lactic-*co*-Glycolic Acid) Nanoparticles and Nanoliposomes for Protein Delivery in Targeted Therapy: A Comparative *In* *Vitro* Study

**DOI:** 10.3390/polym12112566

**Published:** 2020-11-01

**Authors:** Giulia De Negri Atanasio, Pier Francesco Ferrari, Roberta Campardelli, Patrizia Perego, Domenico Palombo

**Affiliations:** 1 Department of Experimental Medicine, University of Genoa, via Leon Battista Alberti, 2, 16132 Genoa, Italy; giulia.denegriatanasio@edu.unige.it; 2 Department of Surgical and Integrated Diagnostic Sciences, University of Genoa, viale Benedetto XV, 6, 16132 Genoa, Italy; domenico.palombo@unige.it; 3 Department of Civil, Chemical and Environmental Engineering, University of Genoa, Via Opera Pia, 15, 16145 Genoa, Italy; p.perego@unige.it; 4 Research Center for Biologically Inspired Engineering in Vascular Medicine and Longevity, University of Genoa, via Montallegro, 1, 16145 Genoa, Italy; 5 Vascular and Endovascular Surgery Unit, IRCCS Ospedale Policlinico San Martino, Largo Rosanna Benzi, 10, 16132 Genoa, Italy

**Keywords:** cardiovascular diseases, protein drug delivery, nanocarriers, polymer-based nanosystem, lipid-based nanosystem, poly (lactic-*co*-glycolic acid), phosphatidylcholine, hemolysis, biocompatibility

## Abstract

Over the previous years, the design, development, and potential application of nanocarriers in the medical field have been intensively studied for their ability to preserve drug properties, especially their pharmacological activity, and to improve their bioavailability. This work is a comparative study between two different types of nanocarriers, poly (lactic-*co*-glycolic acid)-based nanoparticles and phosphatidylcholine-based nanoliposomes, both prepared for the encapsulation of bovine serum albumin as a model protein. Polymeric nanoparticles were produced using the double emulsion water-oil-water evaporation method, whereas nanoliposomes were obtained by the thin-film hydration method. Both nanocarriers were characterized by morphological analysis, particle mean size, particle size distribution, and protein entrapment efficiency. *In* *vitro* release studies were performed for 12 days at 37 °C. In order to explore a possible application of these nanocarriers for a targeted therapy in the cardiovascular field, hemolytic activity and biocompatibility, in terms of cell viability, were performed by using human red blood cells and EA.hy926 human endothelial cell line, respectively.

## 1. Introduction

In recent decades, smart drug delivery systems have received an increasing attention for their ability to locally deliver bioactive molecules to diseased cells, leading to a tangible refinement of current therapeutic protocols. Although cancer therapy still remains the main target of drug delivery carriers, recently they have been taken into consideration even for the treatment of other pathologies.

Cardiovascular diseases (CVDs), i.e., peripheral arterial disease, aortic disease, coronary heart disease, stroke, and transient ischemic attack, are the major causes of death in almost all parts of the world. Besides the traditional therapeutic approaches for the management of CVDs, innovative strategies based on drug delivery carriers are also emerging [1]. Experimental studies involving the delivery of peptides [2], proteins, i.e., growth factors [3], nucleic acids [4], and drugs [5] have been proposed in the recent literature and could be considered as the next-generation treatments for CVDs.

In particular, among the peripheral arterial diseases, atherosclerosis plays a crucial role for its high incidence. This pathology is always related to an inflammation of the arteries caused by the accumulation of low-density lipoproteins (LDL) [6]. The macrophages present at the plaque level possess a primary role as they are responsible for the phagocytosis of oxidized LDL that induce them to become foam cells. Activated macrophages start producing numerous molecules that contribute to trigger the inflammation [7]. Although a complete inhibition of proinflammatory molecules could be beneficial in atherosclerosis, it is necessary to maintain a good balance because their activity is essential for other physiological processes. Therefore, the use of therapeutic proteins, encapsulated and directed to the plaque could represent a good compromise to treat atherosclerosis while maintaining other physiological activities.

However, the efficiency of these emerging therapeutic tools is still low due to the lack of retention of the active molecules, their inactivation during the formulation and delivery processes, especially for protein-based therapies, and also to the low fraction of therapeutics that effectively reach the targeted tissue/organ after their administration. These challenges can be overcome by encapsulating therapeutics in optimized working conditions, within appropriate carriers that protect the encapsulated molecules from any adverse conditions of the external environment, and then release them at the desired site of action at a proper dose [8].

The main factors impacting the performances of delivery systems are the loading capacity, the encapsulation efficiency, the protective behavior, the release properties, and the stability over time [9]. In the cardiovascular field, different kinds of delivery systems have been proposed in the recent literature, such as polymeric nanoparticles [10], calcium carbonate nanoparticles [11], nanoemulsions [12], solid lipid particles [13], dendrimers [14], phytosomes [15], micelles [16], liposomes [17], and microgels [18].

Polymeric nanoparticles (PNPs) have been used as potential carries for several classes of drugs, such as anticancer agents [19], antihypertensive drugs [20], immunomodulators [21], hormones [22], and biological macromolecules as peptides [23], proteins [24], and nucleic acids [25]. Over the years, a great variety of natural and synthetic polymers have been exploited for the preparation of nanoparticles. Among them, poly (lactic acid) (PLA), poly (glycolic acid) (PGA), and their copolymer, poly (lactic-*co*-glycolic acid) (PLGA) have been extensively investigated because of their high biodegradability and biocompatibility [26,27]. Different methods are used for PNP preparation such as nanoprecipitation [28], spray-drying [29], emulsion solvent evaporation [30], and microfluidic systems [31]. However, none of them is preferable because the properties of the obtained nanoparticles can be different. They could differ in size, stability over time, and their effectiveness of drug incorporation depends on the adopted preparation technique. The production method can also affect the kinetic of drug release from particles, which is also another important factor to be taken into account working with nanoparticles. Among the technologies proposed for PNP production, the emulsion evaporation method is the most frequently used, since it allows a better control over particle size distribution, reducing solvent residue and resulting in a high entrapment efficiency [32].

Other suitable carriers for peptides, proteins, and nucleic acids are nanoliposomes (NLPs) [33]. They are vesicles formed by an external lipid double layer and an internal aqueous core, which make them useful for the entrapment, the delivery, and the release of water-soluble, lipid-soluble, and amphiphilic compounds. NLPs have been demonstrated to deliver several substances as imaging agents [34], peptides and proteins [35], low molecular weight molecules [36], and nucleic acids [37]. Several batch-scale and a few large-scale techniques have been reported for liposome preparation giving rise to vesicles with different sizes (ranging from nanometers to several microns in diameter) and different number of bilayers. The most common preparation techniques comprise thin-film hydration, reverse phase evaporation, solvent injection, emulsion solvent evaporation, coacervation, freeze-thawing, sonication, extrusion, and high pressure methods [38,39]. Most of these techniques are not suitable for the encapsulation of sensitive molecules because of their exposure to mechanical stresses (e.g., sonication and high-pressure-based methods), to potentially harmful chemicals (e.g., organic solvents and detergents) or to change in pH during the preparation step. For all these reasons, the thin-film hydration method, which historically was the first proposed for liposome production, still remains the most currently used technique [40].

PNPs and NLPs seem to be good candidates for the delivery of proteins to treat CVDs. However, a systematic comparative study between them is still missing.

In this work, two types of nanoparticles were studied: PNPs using PLGA as polymer matrix were produced via the emulsion solvent evaporation method and NLPs were prepared starting from phosphatidylcholine (PC), following the thin-film hydration technique. Bovine serum albumin (BSA) was entrapped as model protein in these two nanosystems. The prepared nanocarriers were characterized by morphological analyses, particle mean size, particle size distribution, and entrapment efficiency. BSA release kinetic was studied for 12 days at 37 °C for both the nanocarriers. In order to prove a potential application of these nanocarriers for a targeted therapy in cardiovascular diseases, hemolytic activity on human red blood cells and biocompatibility tests on EA.hy926 human endothelial cell line over a period of 72 h were performed.

## 2. Materials and Methods

### 2.1. Chemicals

Polymeric nanoparticles (PNPs) were produced using poly (d,l-lactide-*co*-glycolide) 50:50 (Mw = 54,000–69,000 g/mol) (RESOMER^®^ RG 505, Evonik Industries, Essen, Germany). Poly (vinyl alcohol) (PVA) (Mw = 31,000–50,000 g/mol), bovine serum albumin (BSA), and phosphatidylcholine (PC) from egg yolk for nanoliposomes (NLPs) were supplied by Sigma-Aldrich (Saint Louis, MO, USA). Ethyl acetate and chloroform used during the preparation and all the reagents for cell culture, i.e., Dulbecco’s Modified Eagle’s Medium (DMEM, high glucose w/l-glutamine w/sodium pyruvate), fetal bovine serum (FBS), trypsin, and Dulbecco’s Phosphate Buffered Saline w/o calcium w/o magnesium (DPBS) were purchased from Carlo Erba (Milan, Italy).

The bicinchoninic acid (BCA) protein assay kit (Euroclone, Pero, Italy) was used to quantify the proteins for calculating the encapsulation efficiency (EE) and for the *in vitro* release studies. CellTiter 96^®^ AQ_ueous_ One Solution Cell Proliferation Assay (MTS) for cell viability studies was bought from Promega (Madison, WI, USA).

### 2.2. Preparation of Polymeric Nanoparticles

PNPs were obtained using an emulsion solvent evaporation method based on a water/oil/water (w/o/w) double emulsion technique. First, the water internal phase was prepared by dissolving BSA in Milli-Q water to obtain solutions at different concentrations (30, 35, and 40 mg/mL). Then, the oil phase was prepared by dissolving 100 mg of poly (lactic-*co*-glycolic acid) (PLGA) in 1.0 mL of ethyl acetate until its complete dissolution. The primary emulsion was obtained adding an adequate volume of the water internal phase containing BSA to the prepared oil phase. For each preparation, the water internal phase volume was calculated to achieve a PLGA to BSA ratio equal to 60% (*w/w*). This value was selected on the basis of a previous optimization study aimed at entrapping BSA with high efficiency [33]. The primary emulsion was obtained by ultrasonic process using a Vibra-Cell™ ultrasonic liquid processor (Sonics & Materials, Inc., Newtown, CT, USA) with a 20 kHz ultrasonic generator probe at 70% amplitude in pulsed mode (30 s on and 30 s off) for 1 min, at room temperature (25 ± 2 °C). The water external phase was a PVA (2%, *w*/*v*) water solution. A fixed amount of water external phase of 4.0 mL was then added to the primary emulsion. The w/o/w emulsion was prepared by sonicating the obtained mixture at the same conditions mentioned above. At the end of the second sonication process, the w/o/w emulsion was diluted up to 7.5 mL with the same external phase solution of PVA aforementioned. The obtained emulsion was left for at least 4 h under magnetic stirring at ambient conditions to allow the complete evaporation of the organic solvent.

The obtained nanoparticle solution was then centrifuged three times at 12,984× *g* for 30 min at 4 °C (centrifuge from Alliance Bio Expertise MF-20R, Guipry, France) and the pellet was washed with deionized water to remove the excess of free BSA. After preparation, polymeric nanoparticle suspensions were stored at 4 °C. Empty PNPs (PNP_E_) were also produced following the same procedure described above and used as control during the entire experimentation.

### 2.3. Preparation of Nanoliposomes

NLPs were produced using the thin-film hydration method. Briefly, 500 mg of PC was dissolved in 100 mL of chloroform. Then, the organic solvent was removed using a rotary evaporator (model Laborota 4000, Heidolph, Schwabach, Germany). Previously, BSA was dissolved in Milli-Q water at three different concentrations (3.0, 4.0, and 6.0 mg/mL) and each aqueous solution was used to hydrate the obtained thin-film layer. The volume of water was always selected in order to maintain the ratio between PC and BSA equal to 60% (*w/w*) for comparison purposes with PNPs. The solution was left under magnetic stirring for 3 h at room temperature. Then, it was homogenized for 2 min using the same Vibra-Cell™ ultrasonic liquid processor reported above at the same conditions. The obtained liposome solution was then centrifuged at 12,984× *g* for 30 min at 4 °C three times and the pellet was washed with deionized water to remove the non-entrapped BSA. Liposome suspensions were stored at 4 °C after preparation. Empty NLPs (NLP_E_) were also produced following the same procedure and used as control during the entire experimentation.

### 2.4. Particles Size

Both PNPs and NLPs were characterized using Dynamic Light Scattering (DLS) with a Zetasizer Nano ZS (Malvern Instruments Ltd, Worcestershire, UK) to measure mean diameter (MD) and particle size distribution (PSD) of the obtained carriers. This instrument worked at 25 °C and was equipped with a 5.0 mW He-Ne laser operating at 633 nm with a scattering angle of 173°. For MD, at least three measurements for each sample were performed reporting their mean value ± standard deviation (SD). In the case of PSD, it has been analyzed after particle preparation for each batch.

### 2.5. Scanning Electron Microscopy

PNP and NLP morphology was studied by using a Phenom ProX desktop SEM (Phenom-World BV, Eindhoven, Netherlands). PNP_E_ and NLP_E_ were prepared as described above. Prior to being analyzed, samples were filtered by using 0.22 μm pore size filters. After that, a drop of each preparation was poured over a glass slide and kept at room temperature until the complete evaporation of water. Before scanning electron microscopy (SEM) analysis, samples were sputtered with gold in the presence of argon. At least three different images for each sample were acquired.

### 2.6. Entrapment Efficiency

The BSA entrapment efficiency (EE) was calculated by an indirect method. The amount of encapsulated BSA was calculated after collection of the supernatants. In detail, both PNPs and NLPs after their preparation were centrifuged at 12,984× *g* for 30 min at 4 °C and the supernatants were collected and analyzed in terms of protein content. EE was calculated according to Equation (1):

(1) $$\mathrm{EE}\;\left(\%\right)=\frac{\mathrm{amount}\;\mathrm{of}\;\mathrm{initial}\;\mathrm{BSA}\;-\;\mathrm{amount}\;\mathrm{of}\;\mathrm{free}\;\mathrm{BSA}\;}{\mathrm{amount}\;\mathrm{of}\;\mathrm{initial}\;\mathrm{BSA}\;}\;\times \;100\;$$

Total amount of initial BSA is referred to the BSA initially used during the preparation procedures while free BSA is the protein present in the supernatants after the centrifugation step; i.e., the non-entrapped. BSA was quantified by BCA assay following the manufacturer’s instructions and the absorbance of the samples was read at 562 nm using a microplate reader (Tecan Spark^®^ 20M, Tecan, Männedorf, Switzerland). This analysis was performed in triplicate.

### 2.7. In Vitro Release Studies

The *in vitro* release studies were performed both with PNPs and NLPs. At first, nanocarriers were centrifuged at 12,984× *g* for 30 min at 4 °C with the same centrifuge reported above to remove free BSA. Then, PNPs and NLPs were resuspended in 10 mL of DPBS (pH = 7.4) and stored at 37 ± 2 °C (incubator VWR, Radnor, PA, USA) under constant stirring. For a period of 12 days at a fixed time, samples were centrifuged at 12,984× *g* and an amount of supernatant corresponding to 3% of the total volume was collected and replaced with the same amount of fresh DPBS. In order to calculate the protein concentration, supernatants were analyzed by using the BCA assay as mentioned before. The obtained values were expressed as percentage of released BSA over time and were determined by summing the released BSA mass at each time point. Release studies were performed in triplicate.

### 2.8. Hemolysis

The *in vitro* evaluation of the nanocarrier compatibility with the red blood cells (RBC) is an important preclinical test. Human blood was obtained by healthy volunteers, who have given their consent by an agreement proposed and accepted by a local ethic committee (9 March 2010) in the context of “Centro di Risorse Biologiche”. Furthermore, hemolysis experiments were performed following the Guidelines of the European Community Council in accordance with the Nuremberg Code (Directive 2004/23/EC). Blood was collected into ethylenediaminetetraacetic acid (EDTA) test tubes and centrifuged for 10 min at 867× *g* (centrifuge SL 8, Thermo Fisher Scientific, Osterode, Germany) in order to separate erythrocytes from plasma [41]. Then, the obtained pellets were washed three times with DPBS while supernatants were discarded. At the end of this procedure, erythrocytes were resuspended in DPBS. Different concentrations of nanocarriers were mixed with erythrocytes to have a solution with a final volume of 150 µL. The solutions were then incubated, under agitation at 25 °C (orbital shaker-incubator ES-20, Grant-bio, Grant Instruments Ltd, Shepreth, Cambridgeshire, England) for 2 h and then centrifuged for 5 min at 867× *g* (centrifuge Z 216 MK, HERMLE Labortechnik GmbH, Wehingen, Germany). The obtained supernatants were spectrophotometrically analyzed at 540 nm using the plate reader reported before. The hemolysis percentage was calculated using Equation (2):

(2) $$\mathrm{Hemolysis}\;\left(\%\right)=\;\frac{{\mathrm{Abs}}_{s}{-\mathrm{Abs}}_{n}}{{\mathrm{Abs}}_{p}{-\mathrm{Abs}}_{n}}\;\times \;100\;$$

where:

${\mathrm{Abs}}_{s}$: is the absorbance of the sample;
${\mathrm{Abs}}_{n}$: is the absorbance of the negative control;
${\mathrm{Abs}}_{p}$: is the absorbance of the positive control.

The negative and the positive controls were obtained treating erythrocytes with DPBS and deionized water, respectively [42]. For this test, the concentration of the particles to be used was calculated on the basis of the amount of entrapped BSA. Negative results were approximated to zero in the analysis and statistical studies were performed exclusively for samples showing a hemolysis percentage lower than 5.

### 2.9. Cell Viability

EA.hy926 human endothelial cells (ATCC^®^ CRL-2922^™^) were cultured in DMEM supplemented with 10% (*v*/*v*) of FBS and incubated at 37 °C and 5% CO_2_ until 70% confluency was reached. 4 × 10^3^ cells were seeded in each well of a 96-well plate and incubated overnight before any treatments. Cells were treated with different concentrations (0.1, 1.0, 10, 100, 200, 300, and 500 μg_BSA_/mL) of both empty and loaded PNPs and NLPs. After 24, 48, and 72 h of incubation with PNPs and NLPs, cell viability was quantified by CellTiter 96^®^ AQ_ueous_ One Solution Cell Proliferation Assay (MTS). Daily, the medium of analyzing well was discarded, cells were washed with DPBS, and a mix of fresh medium (100 μL) and reagent (20 μL) was added to each well and incubated for three hours. At the end of the incubation time, the absorbance of the samples was read at 490 nm by using the same plate reader indicated above. For this test, the concentration of the particles to be used was calculated on the basis of the amount of the entrapped BSA. For PNP_E_ and NLP_E_, an equal amount of the BSA-loaded PNPs and NLPs, respectively, was used for each investigated concentrations. Controls were represented by untreated endothelial cells (without nanoparticles). All the experiments were performed in triplicate and results were expressed as a percentage with respect to the control (100%).

### 2.10. Statistical Analysis

All the experiments were done at least in triplicate and the results are expressed as mean values ± standard deviation. Statistical analysis was done by one-way analysis of variance (ANOVA), following Tukey’s HSD post hoc multiple comparison test using Statistica v 8.0 software (StatSoft, Tulsa, OK, USA).

## 3. Results

### 3.1. Production and Characterization of BSA-Loaded Nanocarriers

Both PNPs and NLPs were successfully loaded with BSA with remarkable differences between them. For the production of PNPs, a protocol based on w/o/w emulsion was adopted. The volume of the internal water phase, when this technique is applied, represents an important parameter which affects entrapment efficiency (EE). The required volume of the internal aqueous phase is mainly determined by the solubility of the compound that has to be encapsulated and it influences particle structure and therefore also the EE [43]. In this work, for the preparation of PNPs, the effect of the variation of the internal aqueous phase volume on mean diameter (MD), particle size distribution (PSD) and EE was studied, maintaining equal to 60% (*w/w*) the theoretical BSA loading with respect to PLGA or PC mass. Specifically, for PNPs it was used an internal volume of water equal to 2.00, 1.70, and 1.50 mL and the concentration of BSA solution was 30, 35, and 40 mg/mL, respectively. Particles prepared without BSA (PNP_E_) were considered as control and taken into account for comparison purposes.

**Figure 1:**
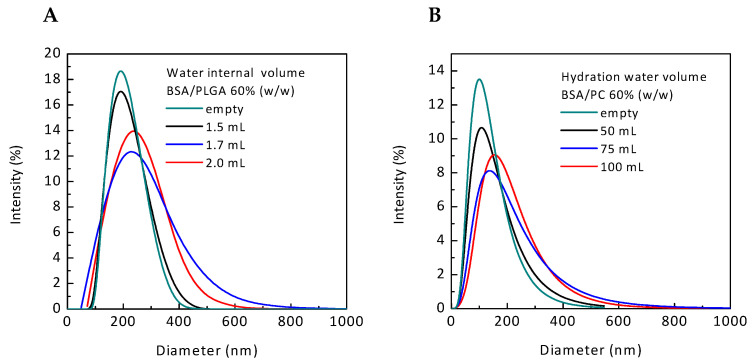
Representative particle size distribution of (**A**) PNPs and (**B**) NLPs. PNPs: polymeric nanoparticles, NLPs: nanoliposomes.

Produced PNPs showed a MD between 170 ± 12 and 204 ± 20 nm. In Table 1 and in Figure 1A it has been reported that particle MD slightly increased at higher water internal phase volumes. Figure 1A highlights also a good control over PSD in all cases, particularly at the lowest water internal phase volume. In the case of PNPs, Table 1 shows a very high EE, up to 98.01 ± 0.05%. The EE slightly increased when the water internal phase volume was reduced. Probably, an increase in the water internal phase volume induces BSA losses towards the external water phase [44]. A higher volume of the internal aqueous phase can induce a decrease in the thickness of the particle polymeric layers allowing the migration of the water internal phase towards the water external phase [45]. The best condition for PNP production with good control of nanoparticle dimensions and high EE was at 1.5 mL of water volume with a BSA concentration of 40 mg/mL.

NLPs were produced using the thin-film hydration method coupled with sonication. In this method, a lipidic layer is produced and then it is hydrated using a water solution containing the molecules of interest, in this case BSA. Spontaneous formation of liposomes is obtained thanks to favorable interactions between water and phospholipids. When liposomes structure is formed, part of the water solution used for hydration is entrapped in the vesicles. The EE is markedly related to the amount of water that is effectively entrapped in the lipid bilayer, with respect to the total amount of solution used for hydration. Therefore, the amount of hydration water is a crucial parameter affecting EE.

For this reason, for NLP production the effects of hydration water volume on liposome MD, PSD and EE were studied. Hydration volume was varied from 50 to 100 mL, changing the BSA concentration from 6.0 to 3.0 mg/mL and keeping constant at 60% (*w/w*) the BSA loading with respect to PC amount. Empty NLPs (NLP_E_), without BSA, were produced for comparison purposes. Operating process parameters and data referred to MD and EE of NLPs are reported in Table 1. It can be observed that NLPs were successfully produced with MD ranging from 130 ± 51 to 144 ± 60 nm by increasing the concentration of BSA in the stock solution used for their preparation. Figure 1B reports obtained PSD of liposomes produced with different water hydration volumes. From Table 1 and from Figure 1B, it can be noticed an increase of liposome MD when higher hydration water volume was used. PSD data, showed in Figure 1B, showed also a good control of liposome dimensions in all the studied cases. Data reported in Table 1 showed remarkable differences in EE values between PNPs and NLPs. The samples produced with 100 and 75 mL of hydration water showed an EE of 46.14 ± 14.17 and 49.49 ± 2.18%, respectively. Conversely, by reducing the hydration volume at 50 mL and using a more BSA concentrated solution in order to have the 60% of theoretical loading, higher EE was obtained (80.16 ± 7.46%). This result is in agreement with the related literature. By increasing the water hydration volume and fixing the PC content, less PC was available for unit of water, reducing the probability of entrapment of the water volume.

The best condition for NLP production with good control over liposome dimensions and high EE was at 50 mL of water hydration volume with a BSA concentration of 6 mg/mL.

Taking into account the two proposed nanosystems, EE was higher in the case of PNPs. The highest EE value (98.01 ± 0.05%) was obtained working with 1.5 mL of water internal phase and a final BSA concentration in the water solution equal to 40 mg/mL. Figure 1A,B report PSD of PNPs and NLPs, respectively. The loading of BSA did not interfere with PSD.

**Figure 2:**
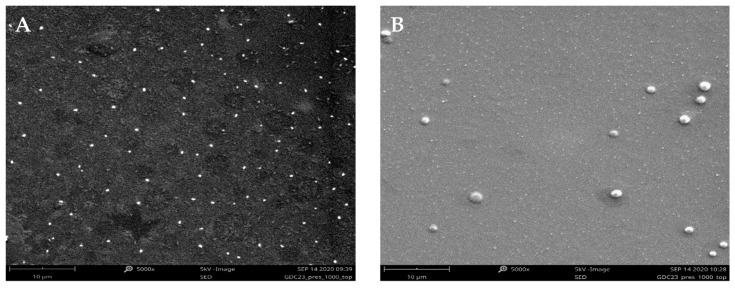
Representative SEM images of (**A**) PNP_E_ and (**B**) NLP_E_. SEM: scanning electron microscopy, PNP_E_: empty polymeric nanoparticles, NLP_E_: empty nanoliposomes.

As reported in Figure 2, the analyses of the morphological properties of empty PNPs and NLPs were performed using SEM. The studied nanocarriers appeared to show a spherical, well-defined morphology.

### 3.2. In Vitro Release Studies

**Figure 3:**
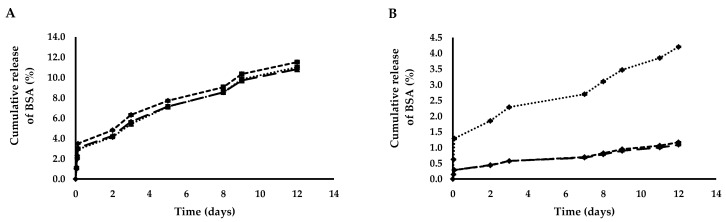
Release profile of BSA from (**A**) PNPs: 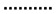 PNP_BSA (30 mg/mL)_, 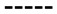 PNP_BSA (35 mg/mL)_, 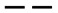 PNP_BSA (40 mg/mL)_ and from (**B**) NLPs: 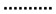 NLP_BSA (3 mg/mL)_, 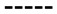 NLP_BSA (4 mg/mL)_, 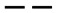 NLP_BSA (6 mg/mL)_. Data are expressed as mean of three measurements. Error bars indicate SD. BSA: bovine serum albumin, PNPs: polymeric nanoparticles, NLPs: nanoliposomes.

*In vitro* release of BSA was studied at 37 °C over a period of 12 days. The three different release curves obtained in the case of PNPs were compared with the three curves obtained working with NLPs. During this time, the maximum amount of BSA released from PNPs and NLPs was 11.01 ± 0.14% and 4.52 ± 0.01%, respectively (Figure 3A,B). This sustained release of the encapsulated bioactive compounds is desirable when they have to reach a pathological site transported by nanocarriers. The delay that is evident in the release of the entrapped molecule is necessary for the nanoparticles to be internalized by the cells. In fact, once they are uptaken by cells, the carriers are able to release the encapsulated molecule that will start its therapeutic effects acting on specific cellular targets. A burst release is not desirable, as it would cause leakage before reaching the target of the pathological site. Working with both the nanoparticles, a burst released of BSA was avoided in all the cases, due to the physicochemical properties of the coating agents, PLGA and PC.

### 3.3. Hemolysis Assay

Measuring hemolysis provides fast and valuable information of the effect of nanocarrier intravenous injections would have on red blood cell (RBC) membrane integrity. All the fabricated particles (PNPs and NLPs) were studied using fixed concentrations of 500, 300, 200, 100, 10, 1, and 0.1 µg_BSA_/mL. The concentration of nanoparticles was considered in terms of BSA content. All the produced samples reported in Table 1 were tested.

**Figure 4:**
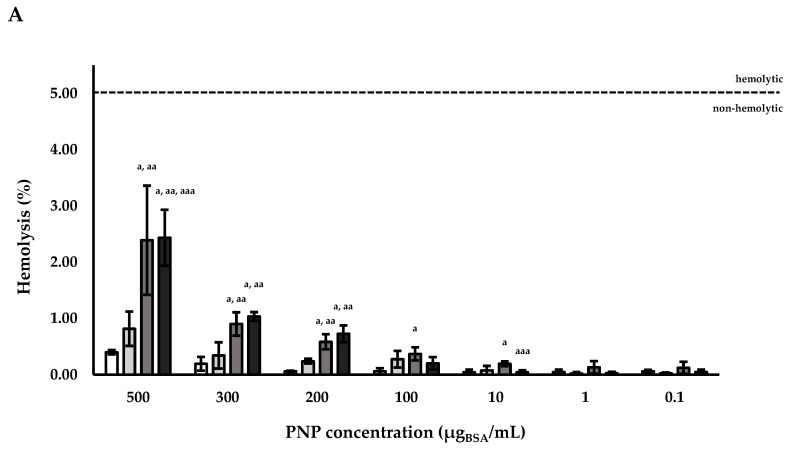

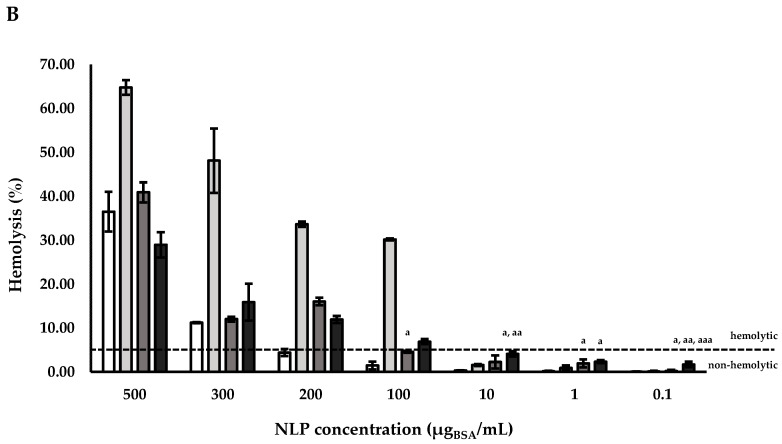
Hemolysis percentage of RBCs after contact with different concentrations of (**A**) PNPs: 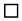
PNP_E_, 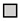 PNP_BSA (30 mg/mL)_, 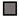 PNP_BSA (35 mg/mL)_, 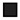 PNP_BSA (40 mg/mL)_ and of (**B**) NLPs: 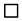 NLP_E_, 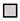 NLP_BSA (3 mg/mL)_, 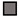 NLP_BSA (4 mg/mL)_, 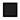 NLP_BSA (6 mg/mL)_. RBCs: red blood cells, PNP_E_: empty polymeric nanoparticles, PNP_BSA_: BSA-loaded polymeric nanoparticles, NLP_E_: empty nanoliposomes, NLP_BSA_: BSA-loaded nanoliposomes. Data are expressed as mean of three measurements. Error bars indicate SD. Different symbols refer to statistically significant differences among results (*p* < 0.05, ANOVA with Tukey’s HSD post hoc multiple comparison test). ^a^: statistically different to empty nanocarriers (PNP_E_ or NLP_E_), ^aa^: statistically different to PNP_BSA (30 mg/mL)_ or NLP_BSA (3 mg/mL)_, ^aaa^: statistically different to PNP_BSA (35 mg/mL)_ or NLP_BSA (4 mg/mL)_. The dotted line refers to 5% of hemolysis.

The obtained results showed that the hemolysis was higher in the presence of NLPs in comparison to PNPs, testing the same concentration. Working with both the studied nanocarriers, there was a direct correlation between the concentration of the payload and the toxicity on the erythrocytes. Figure 4A shows that in the case of PNP_E_, there was a direct correlation between hemolysis percentage and PNP concentrations, especially for the concentration of 500, 300, and 200 µg_BSA_/mL. Regarding the BSA-loaded PNPs, among all the different nanoparticle samples, 500 µg_BSA_/mL resulted to have a higher hemolytic activity in comparison with the same concentration of the PNP_E_ (*p* < 0.05). Furthermore, the hemolytic activity of PNP produced at BSA concentration in the water internal solution of 40 mg/mL (PNP_BSA (40 mg/mL)_) was higher even at the concentrations of 300 and 200 µg_BSA_/mL. For all the tested samples, a hemolytic activity lower than 5% was reported and the formulated PNPs can be considered non-hemolytic at the tested concentrations [46].

Conversely, NLPs showed a hemolytic behavior, even for the empty sample at different concentrations. Concentrations of NLPs equal to 500, 300, and 200 µg_BSA_/mL presented a hemolysis percentage over 5 (Figure 4B). This trend was reported even in the case of BSA-loaded NLPs, but in addition for the loaded liposomes also 100 µg_BSA_/mL concentration presented a hemolysis percentage over 5. The higher values of hemolysis reported for NLPs were probably caused by oxidation of their lipidic layer.

### 3.4. Cell Viability

**Figure 5:**
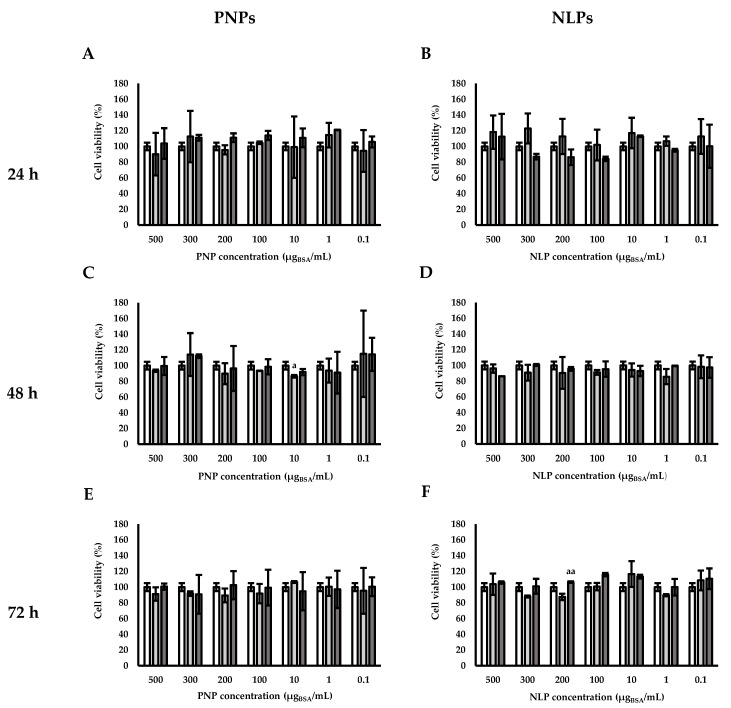
Cell viability of PNPs and NLPs by MTS assay after (**A**,**B**) 24, (**C**,**D**) 48, and (**E**,**F**) 72 h. 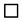 control (without particles), 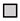 PNP_E_ (**A**,**C**,**E**), 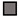 PNP_BSA (40mg/mL)_ (**A**,**C**,**E**), 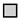 NLP_E_ (**B**,**D**,**F**), 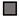 NLP_BSA (3 mg/mL)_ (**B**,**D**,**F**). PNP_E_: empty polymeric nanoparticles, PNP_BSA_: BSA-loaded polymeric nanoparticles, NLP_E_: empty nanoliposomes, NLP_BSA_: BSA-loaded nanoliposomes. Data are expressed as mean of three measurements. Error bars indicate SD. Different symbols refer to statistically significant differences among results (*p* < 0.05, ANOVA with Tukey’s HSD post hoc multiple comparison test). ^a^: statistically different to control, ^aa^: statistically different to empty carrier.

Carriers can be considered as new therapeutic strategy only if they are biocompatible. In this work, biocompatibility was studied in terms of cell viability. Once injected in the body, nanoparticles get immediately in contact with blood and endothelial cells. For this reason, the cell viability of the two studied nanocarriers was assessed on human endothelial cells EA.hy926 by using MTS assay (Figure 5). PNPs obtained with the lower water internal phase volume of 1.5 mL and BSA concentration of 40 mg/mL (PNP_BSA (40 mg/mL)_) were assayed. In the case of NLPs, those obtained with 50 mL of hydration volume and 6 mg/mL of BSA concentration (NLP_BSA (6 mg/mL)_) were chosen. These two samples were chosen since PNP_BSA (40 mg/mL)_ and NLP_BSA (6 mg/mL)_ represented the best compromise between the BSA release and the above-reported hemolytic properties among all the different prepared samples. They represent also the best condition for BSA EE and possess a good control over MD and PSD.

PNPs revealed to have a good biocompatibility for all the tested concentrations (0.1, 1.0, 10, 100, 200, 300, and 500 μg_BSA_/mL) and for all the duration of the experimentation (24, 48, and 72 h). No statistical differences were observed between the untreated (control) and the cells treated with PNPs for all the tested concentrations both for empty and for BSA-loaded nanocarriers. Similar results were obtained working with NLPs. No significant statistically differences were highlighted when comparing cells treated with the same concentration of NLPs over time (24, 48, and 72 h). Furthermore, none of the tested NLP concentration caused any significant statistically decrease in cell viability at each time point. For both PNPs and NLPs, no statistically significant differences were noticed among different concentrations of the same nanocarriers at the same time point, among different time points at the same concentration for and between empty nanocarriers and loaded nanocarriers at a given concentration and a given time-point. The only exceptions were the statistically differences between control and PNP_E_ at 10 μg_BSA_/mL after 48 h of treatment (Figure 5C) and between NLP_E_ and NLP_BSA (6 mg/mL)_ at 200 μg_BSA_/mL after 72 h of treatment (Figure 5F).

## 4. Conclusions

In this work, poly (lactic-*co*-glycolic acid)-based nanoparticles (PNPs) and phosphatidylcholine-based nanoliposomes (NLPs) were produced by a solvent emulsification evaporation method, based on a water/oil/water double emulsion technique, and a thin-film hydration method, respectively. Bovine serum albumin (BSA) was chosen as model protein to be easily replaced by specific therapeutic proteins useful for CVD treatment and was successfully encapsulated in both PNPs and NLPs, varying the volume of the internal aqueous phase. The two studied nanocarriers showed comparable mean size, particle size distribution, and morphological properties in terms of dimension and overall 3D structure. PNPs showed higher entrapment efficiencies presenting a maximum value of 98.01 ± 0.05%. Regarding BSA release, the two studied nanocarriers showed a different release profile: PNPs have released 11.53 ± 0.06% while NLPs have released 4.61 ± 0.02% of the encapsulated BSA after 12 days. In both the cases, considering the amount of released BSA, a burst release of the entrapped protein was avoided. The obtained results show that all the studied concentrations of PNPS have not induced erythrocyte membrane damages. Unlike PNPs, NLPs presented a hemolytic activity at all the concentrations higher than 10 μg_BSA_/mL. Both PNPs and NLPs showed a comparable biocompatibility with human endothelial cells. On the basis of the obtained data, the choice of the better nanosystem strictly depends on the PNPs or NLPs final application. However, the studied nanocarriers can be considered as a good template to be engineered with antibodies on their surface in order to be employed in the vascular field as a nanosystem for protein drug delivery.

Future perspectives of this work will involve the engineering of the produced carriers by the decoration of the particle surfaces with specific antibodies to impart a peculiar targeting for atheromatous sites.

## Figures and Tables

**Table 1:** Particle mean diameter and encapsulation efficiency of BSA-loaded nanocarrier prepared under different conditions. Results are expressed as mean ± standard deviation. Different letters (a, b, and c for PNPs) and (d and e for NLPs) reported in the EE column refer to statistically significant differences among the three different samples for each type of carrier (*p* < 0.05) by ANOVA with Tukey’s HSD post hoc multiple comparison test. BSA: bovine serum albumin, MD: mean diameter, SD: standard deviation, EE: entrapment efficiency, PNPs: polymeric nanoparticles, NLPs: nanoliposomes.

	BSA Concentration (mg/mL)	Water Internal Phase Volume (mL)	MD ± SD (nm)	EE ± SD (%)
**PNPs**	0	1.7	204 ± 20	-
	30	2.0	195 ± 11	97.15 ± 0.07 ^a^
	35	1.7	185 ± 10	97.82 ± 0.07 ^b^
	40	1.5	170 ± 12	98.01 ± 0.05 ^c^
**NLPs**	0	75	130 ± 51	-
	3	100	175 ± 62	46.14 ± 14.17 ^d^
	4	75	152 ± 68	49.49 ± 2.18 ^d^
	6	50	144 ± 60	80.16 ± 7.46 ^e^
